# Why Robots Should Be Social: Enhancing Machine Learning through Social Human-Robot Interaction

**DOI:** 10.1371/journal.pone.0138061

**Published:** 2015-09-30

**Authors:** Joachim de Greeff, Tony Belpaeme

**Affiliations:** 1 Centre for Robotics and Neural Systems, Plymouth University, Plymouth, United Kingdom; 2 Interactive Intelligence Group, Delft University of Technology, Delft, the Netherlands; University of Vermont, UNITED STATES

## Abstract

Social learning is a powerful method for cultural propagation of knowledge and skills relying on a complex interplay of learning strategies, social ecology and the human propensity for both learning and tutoring. Social learning has the potential to be an equally potent learning strategy for artificial systems and robots in specific. However, given the complexity and unstructured nature of social learning, implementing social machine learning proves to be a challenging problem. We study one particular aspect of social machine learning: that of offering social cues during the learning interaction. Specifically, we study whether people are sensitive to social cues offered by a learning robot, in a similar way to children’s social bids for tutoring. We use a child-like social robot and a task in which the robot has to learn the meaning of words. For this a simple turn-based interaction is used, based on language games. Two conditions are tested: one in which the robot uses social means to invite a human teacher to provide information based on what the robot requires to fill gaps in its knowledge (i.e. expression of a learning preference); the other in which the robot does not provide social cues to communicate a learning preference. We observe that conveying a learning preference through the use of social cues results in better and faster learning by the robot. People also seem to form a “mental model” of the robot, tailoring the tutoring to the robot’s performance as opposed to using simply random teaching. In addition, the social learning shows a clear gender effect with female participants being responsive to the robot’s bids, while male teachers appear to be less receptive. This work shows how additional social cues in social machine learning can result in people offering better quality learning input to artificial systems, resulting in improved learning performance.

## Introduction

We argue that social human-robot interaction is an interesting means for extending machine learning. It has been shown that robots can provide social cues to human interaction partners, but the social dimension of human-robot interaction is only now being matched up with machine learning. Usually, isolated aspects of the learning robot are considered, such as the physical design (e.g. [1, 2]) or the learning algorithms (e.g. [3, 4]), instead we consider a holistic view of the learning interaction: from the learning strategies up to the social environment in which the learning is embedded. We report on a human-robot interaction (HRI) experiment in which a social robot acquires the meaning of linguistic labels using a variety of social learning strategies. We report on how the robot learns, and also on how human teachers are sensitive to the robot’s social cues.

### Social machine learning

Learning is essential for the development and maturation of human cognition. While some aspects of human development and learning do not require social input, uniquely human cognitive skills—such as linguistic communication, complex motor skills or abstract reasoning—are almost invariably rooted in social learning. Despite social learning being central to human cognition, its contribution to machine learning remains relatively limited. It is however believed that machines able to take advantage of social learning, by exploiting unstructured social guidance typically offered by humans, will be faster at learning, and will acquire more robust skills.

Children and their carers have a wide range of strategies and motivations which provide a substrate for social learning. For example, infantile facial features positively influence the quantity and quality of care and attention given by adults [5, 6], the presence of turn-taking abilities [7], joint attention [8], imitation skills [9] and associative learning [10] all contribute to language development. It is possible for machines to tap into this human propensity for social tutelage, and a number of promising avenues have been explored. For example, in Learning by Demonstration (also known as imitation learning) the aim of a learning system is to pick relevant elements from one or more demonstrations by a human, and to use these to imitate the goal of the demonstration while generalising to novel situations [9, 11–15]. In most imitation learning implementations, the demonstration process is unidirectional and often uni-modal. Instead social learning in humans relies on bidirectional interaction [16]. The teacher offers tutoring to the learner, and the learner shows how well it has learnt not only through its performance at the task at hand, but also through other modalities, such as non-verbal and verbal expressions. The term *socially guided machine learning* was coined by Thomaz [17] as an umbrella term for a range of approaches in which social interaction between a user and a machine helps to structure the learning input, often to be suitable for classic machine learning [3, 18] or active learning algorithms [19]. In socially guided machine learning, the learning input is often less structured and can be provided by laypersons, who do not require formal training but instead can use natural interaction to teach a machine novel knowledge and skills.

### Human-robot interaction

If a machine—such as a robot—and its behaviour are suitably designed, then people should find it more natural to teach or tutor the machine [20–24]. For this, the interaction should not only be natural, but preferably also desirable. Just as, in most adults, children elicit a response to nurture, care and tutor, we set out to study whether it is possible to design a robot to elicit a similar response.

In this respect social robots provide unique opportunities for the implementation of social machine learning. In contrast to systems such as computers and handheld devices, robots are embodied, operating within the same physical environment as humans and are typically utilising modalities familiar to people, such as language, vision, hearing, touch, facial expressions and gestures. Moreover, social robots often tend to be designed to portray a character, thus stimulating their anthropomorphisation by human interactants and inviting an interaction-style that is natural to people. Both a robot’s appearance and behaviour can strengthen interactants’ interpretation of dealing with a social agent, rather than with a piece of equipment [25]. In this process the careful management of expectations is paramount; when done properly, people’s natural tendencies to anthropomorphise can facilitate and enhance their social interaction with robots [26].

Thus, our working hypothesis is that machine learning might benefit from a social component, and that human-robot interaction provides a natural context for achieving this. Social robots provide a suitable platform on which to implement socially enhanced machine learning. In the following sections we describe in more detail the particular learning task and the implementation on a social robot.

### Learning the meaning of words

Fundamental to human cognition is the ability to use concepts as a means of organising our mental world [27, 28] and linguistic communication [29]. In addition, the formation of concepts (or categories) lies at the heart of the typical machine learning problem of classification—that is, the grouping of external stimuli based on some common feature, usually through descriptive labels—as such bearing considerable similarity to learning the meaning of words. While one might try to draw a distinction between learning the meaning of words and learning concepts, it is generally perceived that, particularly for young children, learning the meaning of a word implies learning the concept that this word signifies [30].

Young children typically acquire new words with remarkable speed [31]. While approaches such as Latent Semantic Analysis [32] have modelled this phenomenon through statistical learning of word co-occurrences, typically the social and semantic context in which words are used is not taken into account. However, it has been shown that children rely on a number of constraints beyond mere statistical properties of word occurrences, significantly aiding them in the otherwise daunting task of correlating their caregivers’ utterances with the correct meaning [10, 33–35]. Furthermore, children do not learn in isolation. They inhabit a rich social environment and substantially aided by capacities such as mutual gaze understanding, shared attention and the notion of others as social beings [36–38]. In addition, computational modelling has shown that aspects of language development can be explained as resulting from mapping multi-modal signals onto each other [39] combined with repeated social interaction between infants and their caregivers, e.g. [40].

### Social learning of word-meaning association on a robot

We describe the design of a robot for social learning of word-meaning associations, and hypothesise that endowing the robot with the ability for social learning not only aids its learning performance but also results in a different attitude by human interactants towards the robot and its learning aims. For this we use a robot which has been designed to evoke a strong social response from adults. We manipulate the social machine learning behaviour on the robot: in one condition the robot is equipped with the ability to use social cues to influence the tutoring of the human teacher and thus enhance the learning input it is offered, while in the second condition it does not possess this ability. We measure whether and to what extent people are sensitive to these cues, and how social cues impact on their tutoring behaviour. The experiment serves as an illustrative case of how the addition of a social component within human-robot interaction may aid in creating a potent learning environment for the robot.

The following sections describe the learning mechanism used by the robot, the participants and materials used, the experimental setup and the metrics. We then discuss the results of the learning experiment, people’s tailoring of the robot’s learning input, a video analysis of the participants’ gazing behaviour and the questionnaire results. This is followed by a discussion on observed gender differences, after which we provide a perspective on how the findings are embedded within social human-robot interaction.

## Methods

The experiment requires a hardware component and a software component: the hardware consists of a social robot and a large 26-inch capacitive touchscreen, while the software consists of a structured interaction between the human participants and the robot (a *language game*) with additional social learning mechanisms.

### Language Games

**Fig 1:**
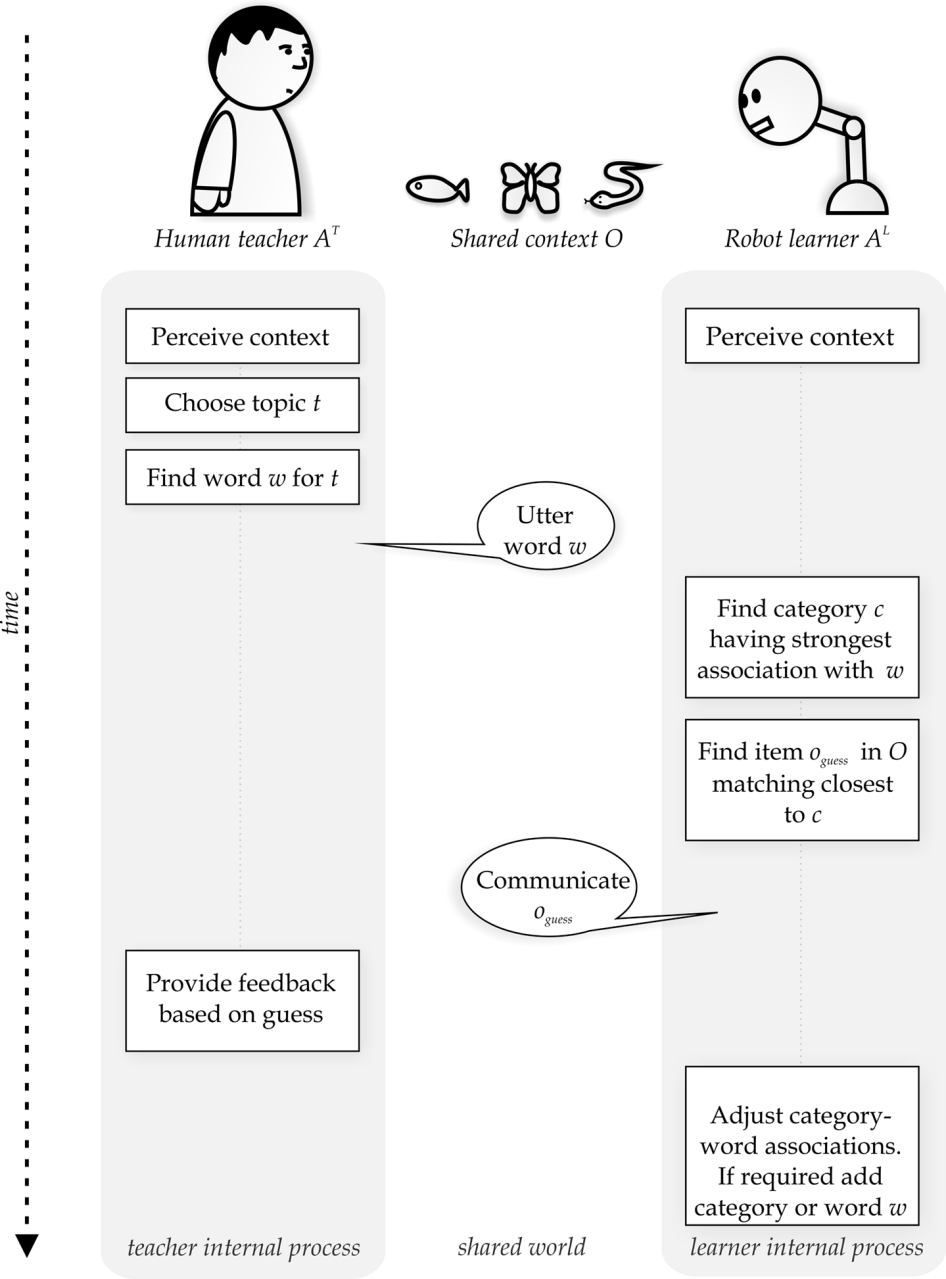
Schematic illustration of the language game flow. Both teacher and learner examine a shared world-view consisting of images. The teacher chooses one image as the topic and communicates an associated linguistic description to the learner. The learner tries to guess which image the teacher has in mind and receives feedback on its guess from the teacher. Based on this feedback the learner modifies its word-meaning associations.

The interaction between human participants and the robot is modelled through a language game [41, 42]. A language game is a single turn in a linguistic interaction and is played between two agents (people or robots). Both agents are presented with a shared world-view called the ‘context’, which consists of images. One agent names an image (the ‘topic’) without revealing which image it refers to and the other agent tries to guess the referent based on the provided name. This interaction is the essence of a single linguistic turn between two language users. When agents repeatedly play language games, it has been shown that both agents can reach an agreement on a lexicon and associated meanings [43–47]. While language games are often used to study the dynamics of language change, they can also be used to model the interaction between a teacher and learner. Through iteratively playing language games with a teacher, the learner will assimilate a lexicon and associated meanings. Fig 1 depicts a schematic overview of a single language game interaction, and a formal description of the language game as used in the experiments is provided below.

The agent stores categories in a Conceptual Space [48], a ℝ^*N*^ space (with *N* being the number of conceptual dimensions) in which each concept takes up a distinct region. Similarity in the Conceptual Space can be calculated as the inverse of the Euclidean distance between two points. Agents can be adaptive: over the course of many games they alter their word-meaning associations depending on the outcome of the games. As such, they eventually learn to ‘speak the same language’. In the case of a teacher-learner game, the learner starts without any knowledge, and only the learner adapts its word-meaning associations based on the teacher’s feedback; the teacher’s knowledge (incorporated as word-meaning associations) remains fixed.

#### Language game algorithm

An agent *A* consists of a Conceptual Space *C* populated with *i* concepts [*c*_1_, *c*_2_, ‥, *c*_*i*_] ∈ *C*, a lexicon *W* containing *j* words [*w*_1_, *w*_2_, ‥, *w*_*j*_] ∈ *W* and an *i* × *j* matrix *M* encoding the associated strength between each *c* ∈ *C* and *w* ∈ *W* as a scalar [0.0,1.0]. An agent *A* ^*T*^ acts as a teacher and an agent *A* ^*L*^ acts as a learner. *A* ^*L*^ starts with *C* ^*L*^, *W* ^*L*^, *M* ^*L*^ = ∅, while *C* ^*T*^, *W* ^*T*^, *M* ^*T*^ are typically populated with predefined domain knowledge.

1. Both *A* ^*T*^ and *A* ^*L*^ observe a shared world, called the context *O*, containing *n* objects [*o*_1_, *o*_2_, ‥, *o*_*n*_] ∈ *O*; the dimensionality of *O* matches the dimensionality of *C*.
2. During each round, *A* ^*T*^ chooses one object $o_{t}^{T}\in O$ as the topic for the game, projects $o_{t}^{T}$ into *C* ^*T*^, finds through similarity measurement the closest matching $c_{t}^{T}$ and communicates the strongest associated word $w_{t}^{T}$ to *A* ^*L*^.
3. *A* ^*L*^ hears $w_{t}^{T}$ as $w_{t}^{L}$, finds concept $c_{t}^{L}$ with the strongest association and assigns the closest matching *o* as $o_{t}^{L}$. If *C* ^*L*^ = ∅, *A* ^*L*^ creates a new $c^{L}=o_{t}^{T}$ and associates this with $w_{t}^{T}$ with default strength 0.5, after which the round ends.
4. If $o_{t}^{T}=o_{t}^{L}$ the game is (a) a success, otherwise it (b) fails. The outcome is indicated by *A* ^*T*^, after which *A* ^*L*^ updates its *C* ^*L*^, *W* ^*L*^ and *M* ^*L*^ as follows:
  (a) *Success*: *A* ^*L*^ increases the association *m* ^*L*^ between the word used to describe the topic ($w_{t}^{L}$) and its internal concept ($c_{t}^{L}$) that led to the guess with 0.1. In addition $c_{t}^{L}$ is modified by including the features from $o_{t}^{L}$.
  (b) *Failure*: *A* ^*L*^ decreases the association *m* ^*L*^ between the word used to describe the topic ($w_{t}^{L}$) and its internal concept ($c_{t}^{L}$) with 0.1. In addition either the internal concept that best matches $o_{t}^{T}$ (which may or may not be $c_{t}^{L}$) is modified by including the features from $o_{t}^{T}$, or a new internal concept is created using the features from $o_{t}^{T}$ and associated with $w_{t}^{L}$. Which option is chosen—modifying an existing concept, or creating a new one—is based on the ability of *A* ^*L*^ to uniquely discriminate $o_{t}^{T}$ from *O*; if this is below a parameterised threshold, a new concept is created.

In other words, after correctly guessing a particular image based on the word provided by the teacher, the learner strengthens the association between this word and its internal concept that best matched the image, while in addition the internal concept is adjusted by incorporating features from the image.

When the learner fails to point out the image matching the word used by the teacher, the association between that word and the internal concept from the learner that best matched the chosen image is decreased. In addition, the learner modifies its internal concept that best matches the actual topic of the game to include features of this image, or it creates a new internal concept with the features of this image, and associates this new concept with the word that the teacher used. By playing multiple language games, the effect of the learning mechanism is that the learner gradually adapts both its set of internal concepts and the associated words to better reflect the knowledge provided by the teacher.

The context that is used in the experiment consists of exemplar animals belonging to the categories ‘mammal’, ‘bird’, ‘reptile’, ‘invertebrate’, ‘insect’, ‘fish’ and ‘amphibian’. The participant, in the role of teacher, mentally chooses one animal out of a set of three as the ‘topic’ and provides the corresponding category label. The robot then tries to guess what the topic is—that is, which animal does the teacher refer to—based on the category label provided. The animal categories are gradually learned by the robot through the incremental refinement of the associations between words and meanings based on the teacher’s feedback, as described above. The choice of topic, i.e. which animal categories to teach, is left free for the teacher to choose. Success and failure rates are recorded for each round, providing a measure of how well the robot is able to make the correct guess over the course of interactions.

### Participants

A total of 41 participants were recruited from around a British university campus; they received £7.50 for their participation. Ethical approval was obtained from the University of Plymouth faculty of Science and Technology Human Ethics Committee and participants gave written informed consent prior to the experiment. Due to technical failure of the robot during interactions, 2 participants were dropped from the pool and due to poor visibility of recorded video data another participant had to be excluded. Thus, the analysis is based on a total of 38 participants. Participants were randomly assigned to a ‘social’ and a ‘non-social’ condition (see below). The breakdown in terms of native English speakers, gender and age is provided in Table 1.

**Table 1:** Participants.

	social	non-social	total
number	19	19	38
native speaker	13	15	28
non-native speaker	6	4	10
female	9	11	20
male	10	8	18
age (mean)	24.26	24.74	24.5 (SD: 5.03)

Statistical breakdown of participants regarding native language, gender and age.

### Materials

**Fig 2:**
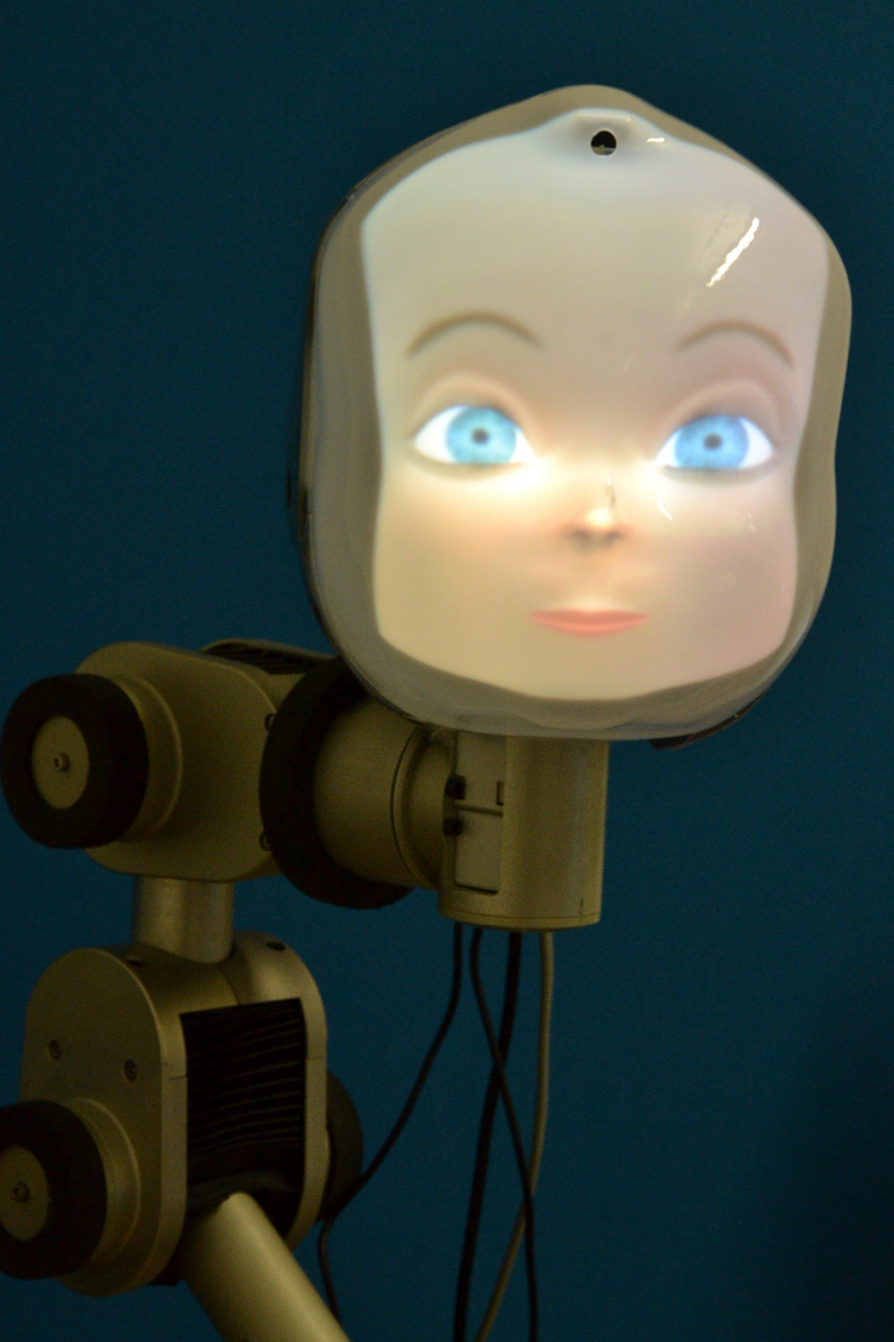
The LightHead robot. A semi-transparent mask mounted on a 6 DoF robotic arm, in which an animated character is projected.

The role of the learner was fulfilled by a robot consisting of a robot head mounted on an articulated robot arm. The head consists of a semi-transparent face, a digital projector (Microvision ShowWX+, 15 lumen) which projects an animated character and lens optics to stretch open the projected image [49]. The face is generated using a 3D graphics model, which contains a model of facial muscles and their interactions. The computer generated face allows the robot to display a wide range of real-time facial expressions. The head is mounted on a Neuronics Katana robot arm, a 6 degree-of-freedom articulated robotic arm, which acts as a neck and spine. The head also has an integrated camera, which is used to record video data and track people near the robot. In addition, the robot is equipped with microphones and a speaker for audio communication (Fig 2).

**Fig 3:**
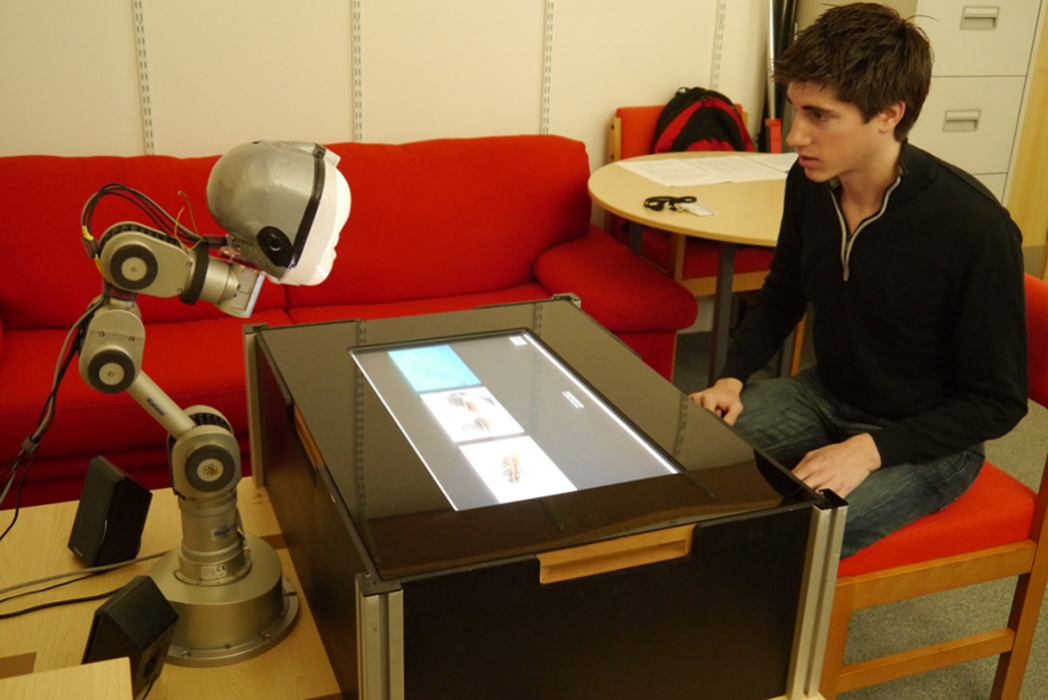
Overview of the experimental set-up, showing the LightHead robot, the touchscreen used to play an interactive learning game and the participant. The individual in this image has given written informed consent (as outlined in PLOS consent form) to publish these case details.

During the game, participants were seated across the robot, with the touchscreen in between that displayed images of animals and seven potential category labels (Fig 3 [50]). The animals were drawn from the Zoo dataset (UCI Machine Learning Repository [51]), which contains 100 animal exemplars belonging to 7 different categories. Animals are encoded through 15 Boolean-valued attributes such as ‘hair’, ‘feathers’, ‘aquatic’ etc. and 1 numerical-valued attribute ‘number of legs’. For each of the animals, an image (found through Google image search) was displayed and both the robot and the participant were shown these images during the experiment.

Unbeknownst to the participants the robot did not perceive the animal based on the image, as the current state-of-the-art computer vision is not able to extract all 16 descriptive attributes from a photo or image alone. Instead, the animal attributes were encoded as a ℕ^16^ vector and fed directly into the robot’s learning mechanism. To the participant however, it appeared as if the robot was examining the images through its eyes and camera, thus creating an illusion of that the robot was visually aware of the learning setup. The camera mounted in the robot forehead was instead used to track the participant’s face, allowing the robot to address the participant and thereby enhancing the social contingency of the interaction. The robot did not rely on speech recognition, as state-of-the-art speech recognition technology is not 100% reliable and could introduce a confound in the experiments. Participants interacted with the robot through tapping words on the touchscreen. We circumvented issues of noisy perception (vision and speech) in this manner, as addressing these was outside the scope of this study.

Data was recorded through the interaction logs, the robot’s camera feed, an additional camera placed behind the robot (to capture participants’ gazing behaviour, which could not be captured by the robot’s camera when the robot was looking down) and a questionnaire administered after the interaction.

### Experimental setup

Participants started with a series of practice rounds in which colour categories were taught to the robot. These practice rounds allowed the participants to become familiar with the robot and the interaction dynamics. After a fixed number of practice rounds (15), or when the participant felt confident enough, they started with the experiment sessions which involved the animal stimuli. Each participant played 50 guessing games with the robot. Simulated language games may typically last for thousands of iterations. Because this is not feasible with human participants, we opted for an acceptable number of games (50), during which the robot can still reach a reasonable performance, allowing us to compare the two conditions. For each guessing game round, the robot’s behaviour—expressed through the robot’s neck movements, facial animation and speech—consisted of the following steps:

1. Verbally announce the next round (e.g. “let’s do another round”).
2. Examine the images of animals by leaning over the touchscreen and fixating gaze on each image in turn.
3. Move to neutral (upright) position.
4. *Social condition only*: express learning preference by fixating gaze on the preferred animal image while simultaneously uttering a statement of interest.
5. Wait for participant to press a linguistic category button.
6. Show guess by leaning over the screen, fixating the gaze on guessed animal and verbally asking whether or not the guess was correct.
7. Depending on the outcome (guess was correct or incorrect), express joy or sadness through a verbal and facial expression.

Two conditions were tested. In the first condition, dubbed ‘non-social’, the robot interacted with the participants according to the scripted behaviour described above, which implemented the learner role of the guessing game. In the second condition, dubbed ‘social’, the robot followed the same script, but in addition tried to convey learning preferences by utilising social cues (step 4 in the behavioural list above, between step 2 and 3 in the language game algorithm description). These social cues consisted of gaze fixation onto a particular animal image that was the robot’s preference and uttering a phrase such as “I would like to learn this one” or “This one looks interesting”; this phrase was picked randomly from a set of 12 possible phrases. The robot’s gazing and the uttering the verbal statement were executed simultaneously, as to provide a multi-modal expression of interest. In contrast, in the non-social condition, the robot would not express any preference for a particular image. It is worth clarifying that in both conditions the robot exhibited behaviour that can be classified as ‘social’, e.g. using verbal interaction (step 1, 6, 7) and expressing joy or sadness depending on the outcome of the game (step 7). However, these social behaviours were fixed for both conditions. Only in the social condition the robot utilized an additional multi-modal social cue (gaze and verbal statement) to express its learning preference, thus modulating the interaction.

The robot’s learning preferences were generated through a mechanism which favours images that are relatively less familiar to the robot. This is done by examining all images and calculating their distance to known concepts residing in the robot’s conceptual space. The object with the greatest distance to a known concept—either a novel animal category, or an exemplar with features that are relatively atypical for its category—becomes the robot’s preference. This can be viewed as akin to the well-known phenomenon of novelty preference in young children [52]. Thus, in the context of the guessing game, the robot examines the images from the context, and the one that is least familiar—based on what is already learned—becomes the robot’s preferred topic. The robot then tries to convey this preference for a particular image by utilising social cues as described above.

It was previously established in simulations that a learner that is actively influencing the teachers’ choice of guessing game topic can enhance its learning experience, both in terms of learning speed and quality; average increase of guessing game success was about 10% [53]. These simulations however, constitute an ideal case in which the teacher always follows the preference of the learner. Whether or not this effect can also be achieved in a real-life human-robot interaction in which the robot relies on conveying its preference through social cues, is the topic of investigation.

### Metrics

The following metrics were used to compare the two conditions:

Robot learning performance This is a measure of the robot’s success in correctly matching the animal class to an animal. For example, the robot picking a picture of a bear from between a lobster and a butterfly when the participant asked to show it a mammal. The performance *P* is calculated as the number of correct guesses *G*_*correct*_ divided by the total number of guesses *G*_*total*_ the robot has made up to that point Eq (1).
  $$\begin{array}{c} P=\frac{G_{correct}}{G_{total}} \end{array}$$ (1)
Participants’ choice of topic During each guessing game, the participant chooses one out of three animals as the topic for the game. There are no constraints on this choice, but it may potentially be influenced by the robot’s social behaviour. The choice of topic was measured during the game, while in addition participants were quizzed on their motivations afterwards, thus allowing for quantitative and qualitative analysis of participants’ choices.
Participants’ gazing behaviour As the analysis of human gazing behaviour may convey important cues regarding a person’s attention [54] and engagement with a robot [55], video recordings of the interaction were coded for the participants’ gazing behaviour (looking at the touchscreen or looking at the robot).
Questionnaire Participants were asked to answer a number of questions addressing their subjective experience regarding their interaction with the robot; in addition, to gain insights into potential influences of their individual personalities on the interaction, they were asked to complete a personality test based on the Big Five Inventory [56].

## Results

### Robot learning performance

**Fig 4:**
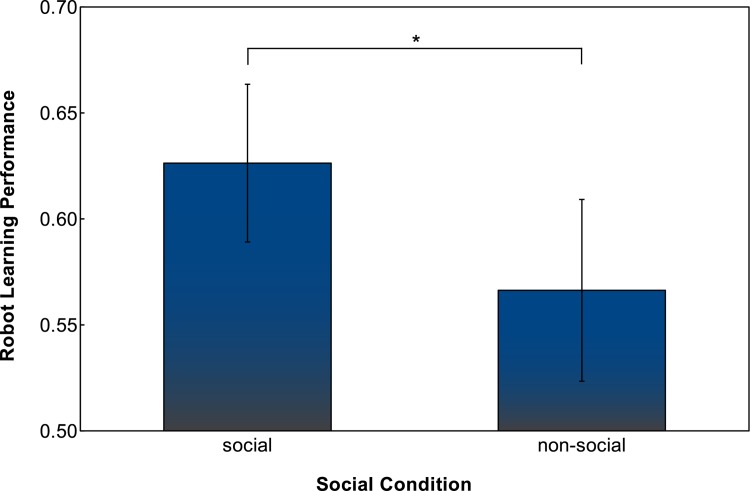
Difference in robot learning performance between social and non-social group. Error bars indicate 95% confidence interval, ‘*’ indicates a significant difference with *p* = 0.0328, two sample t-test.

In the non-social condition average robot performance at the end of interactions was 0.566 (*SD* = 0.089), compared to 0.626 (*SD* = 0.077) in the social condition (Fig 4). There is a small but significant effect of the social behaviour of the robot on the learning performance, which is consistent with the 10% increase found in simulation [53]. This is also confirmed by a two sample t-test with *t*(36) = 2.2206, *p* = 0.0328; the effect size is 0.72 (Cohen’s *D*).

### Choosing a topic

Participants were free to choose the topic of each guessing game (i.e. which animal to teach); to analyse whether or not their choices were influenced by the behaviour of the robot, their teaching behaviour is examined on three different aspects: 1) the distribution of category choices, 2) responsiveness to the robot’s social cues and 3) a qualitative analysis of participants’ category choices.

#### Distribution of participants’ category choices

Comparing the distribution of tutoring examples used by the participants to the distribution of animals in the training data provides some insights into whether or not participants were choosing training examples at random. In the dataset used, the number of animal exemplars for each category are not equal; for instance, there are many more mammals (40) than birds (20) or reptiles (5). As such, if participants chose a random animal as the topic, the aggregated distribution of category choices is expected to be very close to the distribution of the database. However, this is not what was observed. Statistically significant differences between the dataset distribution and the distribution of participants’ choices were found for the reptile, invertebrate, insect, amphibian and mammal categories in the non-social condition, and for the reptile, fish, invertebrate, insect and mammal categories in the social condition.

**Fig 5:**
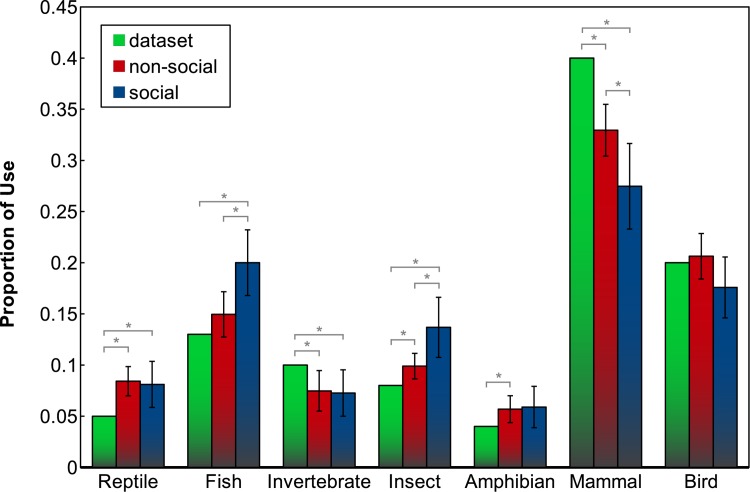
Distribution of participants’ category choices in social and non-social condition, compared to the dataset distribution. Error bars indicate 95% confidence interval.

As illustrated in Fig 5, it is clear that participants diverge from the database distribution, both in the social and the non-social condition. In other words, they do not choose tutoring examples randomly, but follow a certain strategy (which may or may not be conscious). People tailor the learning input for the robot, even if it is not actively soliciting this, as is the case in the non-social condition. However, their diverging from the dataset distribution is more pronounced in the social condition; the difference in category use between social and non-social condition is significant for the fish, insect and mammal categories (two-sided t test with *t*(36) = 2.5385, *p* = 0.0156, *t*(36) = 2.3233, *p* = 0.0259 and *t*(36) = −2.1935, *p* = 0.0348 respectively), see Table 2. This illustrates that the additional social cues employed by the robot in the social condition can influence participants’ choice of topic.

**Table 2:** Comparison of participants’ choices of animal types as percentage of the whole of their interaction against database distribution.

category	database	non-social mean (SD)	social mean (SD)	t test	
reptile	0.05	0.0842 (0.03)	0.0810 (0.05)	t(36) = -0.2322,	p = 0.8177
fish	0.13	0.1495 (0.05)	0.2000 (0.07)	t(36) = 2.5385,	p = 0.0156 *
invertebrate	0.1	0.0747 (0.04)	0.0726 (0.05)	t(36) = -0.1369,	p = 0.8918
insect	0.08	0.0989 (0.03)	0.1368 (0.07)	t(36) = 2.3233,	p = 0.0259 *
amphibian	0.04	0.0568 (0.03)	0.0589 (0.04)	t(36) = 0.1711,	p = 0.8651
mammal	0.4	0.3295 (0.06)	0.2747 (0.09)	t(36) = -2.1935,	p = 0.0348 *
bird	0.2	0.2063 (0.05)	0.1758 (0.07)	t(36) = -1.6103,	p = 0.1161

Comparison of the distributions of participants’ choice of animal categories for the non-social condition and social condition. A two-sample t test compares the means from both conditions for each animal category. Significant differences are indicated with ‘*’.

The fact that participants appear to tailor the diet of tutoring examples, both in the social and the non-social condition, might be explained as people forming a “mental model” of the robot. A mental model is a cognitive process in which a person forms expectations about beliefs and goals of another agent, be it a person or a machine. Mental model forming does not need to be conscious, Byron and Nass for example argues that people treat machines as being human-like and require a mental model of the machine to do so [57, 58]. This has e.g. been shown to arise from the robot’s physical attributes [1]. Here we suggest that in addition to the physical character of the robot, its behaviour also might influence mental model forming. The corollary here being that people are better at training social robots and machines in an interactive manner, because of the mental model they form of the learning process which allows them to feed the robot an optimised training set [3, 21].

#### Responsiveness to the robot’s social cues

**Fig 6:**
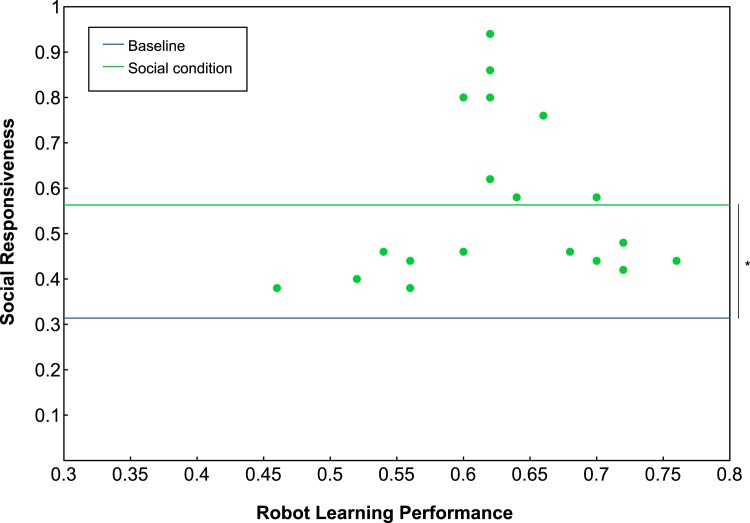
Social-responsiveness. The responsiveness to the robot’s social cues plotted against the robot learning performance. Straight lines depict a 33% baseline of following the robot’s preference by chance (blue) and the mean value of social responsiveness (green).

To quantify the extent with which participants were responsive to the robot’s social cues, we define *social-responsiveness* as the number of times the teacher’s exemplar choice matched the preference of the robot, divided by the total number of guessing games played. In the non-social condition, the robot did not calculate a preferred topic, so we compare the social responsiveness of participants in the social condition to a 33% baseline of following the robot’s preference by chance. In the social condition, it is clear that participants to various degrees adhere to the robot’s preference. Mean social-responsiveness in the social condition is 56.3% (*SD* = 18%), which is significantly different from chance (one-sample t test with *t*(18) = 5.5645, *p* < 0.0001), see Fig 6. Furthermore, the figure depicts the social-responsiveness for each participant against the robot learning performance in the social condition. What can clearly be observed is the general tendency of high social-responsiveness combined with a relatively high robot learning performance. It is also clear though that a high social-responsiveness does not guarantee a high robot learning performance; indeed, only a weak correlation was found between the two (social condition, Pearson’s *r* = 0.09).

#### Qualitative analysis of participants’ category choices

The questionnaire asked participants to indicate what motivated their choice for a particular animal as the topic for the guessing game (“On what basis did you choose the animal examples as topic? Please explain.”). This question was open, so a variety of answers were given. Some participants stated they made their choices based on whether or not they liked the animals, others tried to use a variety of different categories while yet others mentioned to base their choice (somehow) on the learning robot. We grouped these answers into the following seven types of motivation: “1: animal categories (e.g. choice based on different categories, reproductive system, etc.)”, “2: robot learning considerations (e.g. making it easy or hard for the robot to learn)”, “3: robot’s preferences (choosing the topic based on the robot’s social cues)”, “4: personal knowledge (e.g. choosing the type of animal that the participant was familiar with)”, “5: personal preference (e.g. choosing an animal based on how much the participant liked/disliked a particular animal)”, “6: random (explicitly stating the choice was random)” and “7: other (e.g. first thing that came to sight)”. Table 3 displays the total numbers for this grouping.

**Table 3:** Participants’ motivation for choosing a particular animal.

Motivation for choosing a particular animal	total	NS	S	F-NS	M-NS	F-S	M-S
1: biological classification (general knowledge)	3	2	1	1	1	0	1
2: robot learning considerations (easy/hard)	14	7	7	4	3	2	5
3: robot’s preferences (e.g. looking behaviour)	4	0	4	0	0	3	1
4: personal knowledge (familiarity with animals)	13	5	8	3	2	6	2
5: personal preference (liking/disliking animals)	10	7	3	6	1	2	1
6: random	3	2	1	1	1	0	1
7: other	1	1	0	1	0	0	0

Participants’ motivation are grouped into motivation types. Data is split into social (S) and non-social (NS) groups, as well as gender (F-NS, F-S, M-NS and M-S).

As expected, in the social condition, some participants explicitly mention their choice being motivated by the robot’s preference. In addition, participants in the social condition report having based their choice more on their knowledge of the animal classification (motivation 4), which contrasts with the non-social condition in which participants’ choice appears to more motivated by personal preferences (motivation 5). Interestingly, for the female participants a large shift can be observed from personal preference (motivation 5) in the non-social condition to personal knowledge (motivation 4) in the social condition. For male participants, in the social condition, robot learning considerations as a motivation increase. However, based on a Fisher’s Exact Test we cannot rule out the possibility that the observed patterns in the answers are due to chance (*p* = 0.222).

### Video analysis of participants’ gazing behaviour

During the interaction, video recordings were made using a camera placed just behind the robot’s head. These recordings were coded for the participants’ gazing behaviour, for which three categories were defined: “looking at the robot” (LR), “looking at the touchscreen” (LT) and “looking elsewhere” (LE). Coding of all interactions was performed by one coder, and a random subset of 10% of the interactions was coded by a second coder which resulted in an inter-coder agreement of 85% (with Cohen’s Kappa *K* = 0.72, indicating good agreement).

Participants’ looking behaviour turned out to hardly fall in the LE category (< 1%), hence the LE category is omitted from the analysis. Depending on the social condition, there was a difference in robot behaviour. In the social condition the robot uttered an extra verbal statement in order to convey its learning preference, while in addition repeatedly looking back and forth between the participant and the animal of its preference. In the non-social condition, the robot examined the animals on the screen and after that kept looking at the participant.

As can be observed in Table 4, participants may have responded to the robot’s social cues by altering their gazing behaviour, as in the social condition they spent a larger percentage of the time looking at the robot (not significant, but indicating a trend). In addition, there appears to be a trend of increased gaze changing between robot and touchscreen in the social condition (as measured in # gaze changes per minute). Some small differences in gazing behaviour split on gender can be observed as well, but these are also not significant.

**Table 4:** Participants’ gazing behaviour.

Response variable	unit	social	non-social	t test	
LR percentage	percentage	32.98	28.20	t(35.402) = 1.571,	p = 0.1251
LT percentage	percentage	66.50	71.54	t(35.313) = -1.6659,	p = 0.1046
change rate per minute	# changes	11.44	10.00	t(31.396) = 1.7917,	p = 0.0828

Aggregated numbers of participants’ gazing behaviour (gazing at the robot (LR) and gazing at the touchscreen (LT)) based on video analysis; mean values are shown for social and non-social condition.

As such, while participants’ gazing behaviour seems to differ somewhat between conditions, the robot’s social behaviour appears not to have a significant impact. It is quite likely however, that participants are sensitive enough to pick up the robot’s social cues without having to explicitly ‘stare’ longer at the robot. Video analysis of people’s gaze may therefore be too crude a tool to pick up the (if any) subtle behavioural chances that participants might have displayed.

### Questionnaire

Through a questionnaire participants reported on various aspects of their experience after the interaction with the robot. Questions that were asked were e.g. “How do you rate your interaction with the robot?”, “Who was in control of the teaching sessions?” and “How smart do you think the robot is?” (the full list of questions is included as S2 File). The aim was to gain insights in participants’ subjective experience and to assess to what extent the robot’s social behaviour might have influenced this. As it turns out, no significant differences were found between the answers given by participants in the social condition compared to the non-social condition. This was somewhat contrary to our expectations. However, when taking participants’ gender into account, some differences can be observed; this is described in more detail in the section below.

In addition, to identify whether or not certain personality types might be more or less receptive to the robot’s social cues, a personality test based on the Big Five Inventory (BFI) [56] was conducted (see S2 File). The personality test asked participants a number of questions about their personality on a five-point Likert scale (ranging from strongly disagree to strongly agree). Answers to these questions were then translated into a score for the five personality traits addressed by the BFI: openness, conscientiousness, extraversion, agreeableness and neuroticism.

Table 5 displays the correlation scores (Pearson’s *r*) between participants’ BFI personality traits and the robot learning performance and social-responsiveness (for the social condition only). There exist a medium correlation (Pearson’s *r* = 0.4238) between participant’s conscientiousness and their social responsiveness, which is however not significant (*t*(17) = 1.9294, *p* = 0.0705). Other than that the personality test did not reveal any strong correlations between BFI personality traits and either robot learning performance or social-responsiveness. As such, we conclude that the personality type of the participants did not significantly influence the effectiveness of the robot’s social behaviour, although a person’s conscientiousness may play some role.

**Table 5:** Correlation between participants’ personality traits and robot learning performance and social-responsiveness.

	Robot learning performance	Social-responsiveness (social condition)
Agreeableness	-0.0044	-0.0863
Conscientiousness	0.0579	0.4238
Extraversion	0.1628	0.0997
Neuroticism	-0.0402	-0.0325
Openness	-0.2346	0.2439

Correlation (Pearson’s *r*) between participants’ personality traits and robot learning performance and social-responsiveness. Social-responsiveness is only correlated in the social condition, as in the non-social condition the robot did not exhibit social cues and therefore the measurement has no meaning.

### Gender differences

Gender differences are often observed in HRI [59–62], both with respect to the subject’s gender and the portrayed robot gender. The LightHead robot used in our experiment was designed to be child-like and gender-neutral; its name, appearance, voice and behaviour do not contain characteristics that are typically associated with either male or female traits. While it was not formally tested whether or not people perceive the robot to be gender-neutral, an informal survey conducted during a robot exhibition at the London Science museum suggests this to be the case. Out of 114 comments from the public about the LightHead robot’s appearance and behaviour, 23 comments can be interpreted as indicating the LightHead’s appearance to be child-like; descriptions that were used were ‘baby’ (8), ‘doll’ (4), ‘Casper’ (the ghost) (4), ‘child’ (3), ‘not adult’ (2), ‘toddler’ (1) and ‘Chucky’ (1). While ‘Casper’ and ‘Chucky’ may have some boy-like connotations and are as such not gender-neutral, the other 18 comments are. In contrast, none of the comments mentioned the robot appearance to be either male or female. As such, the assumption has been that participants in the experiment predominantly will have perceived the robot as child-like and gender-neutral. However, it might still be the case that the participants’ gender had an influence on the manner in which they viewed the robot and responded to its social cues. To assess this, we analysed the results regarding influences of participants’ gender.

**Fig 7:**
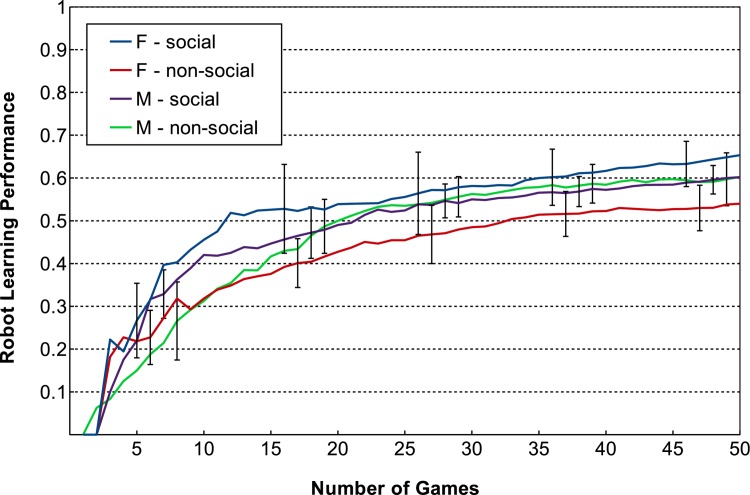
Mean trends of robot learning performance; data is split based on gender and social condition. Error bars indicate 95% confidence interval.

**Fig 8:**
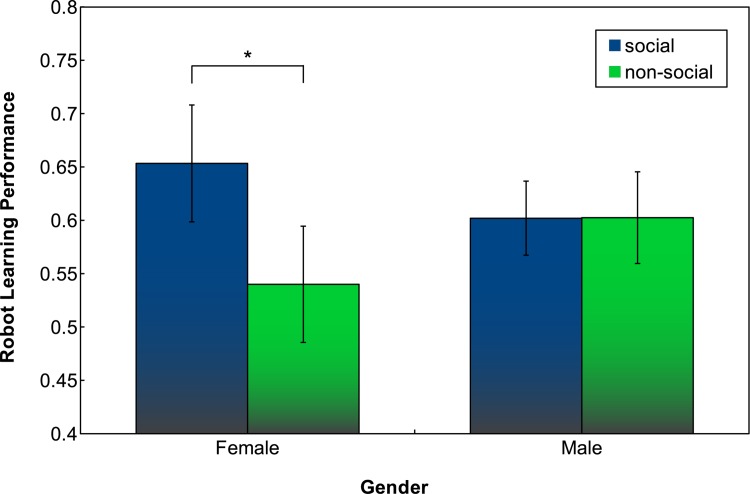
Mean trends of robot learning performance at the end of interacting with the robot. Numbers are split based on gender and social condition, error bars indicate 95% confidence interval.

For the robot learning performance, when splitting the results based on participants’ gender, we found differences between female and male participants. In particular, females appear to be more effective in the social condition compared to males, while in the non-social condition the reverse was observed. This was confirmed through an ANOVA, which indicated a significant interaction between social condition and gender (*F*(1, 34) = 4.7088, *p* = 0.0371); a two sample t-test showed a significant difference in mean robot learning performance for female participants depending on the social condition, with *t*(17.712) = -2.7258, *p* = 0.0140. Fig 7 illustrates the trend of robot learning performance over the course of interaction, while Fig 8 shows the robot learning performance at the end of the game.

**Fig 9:**
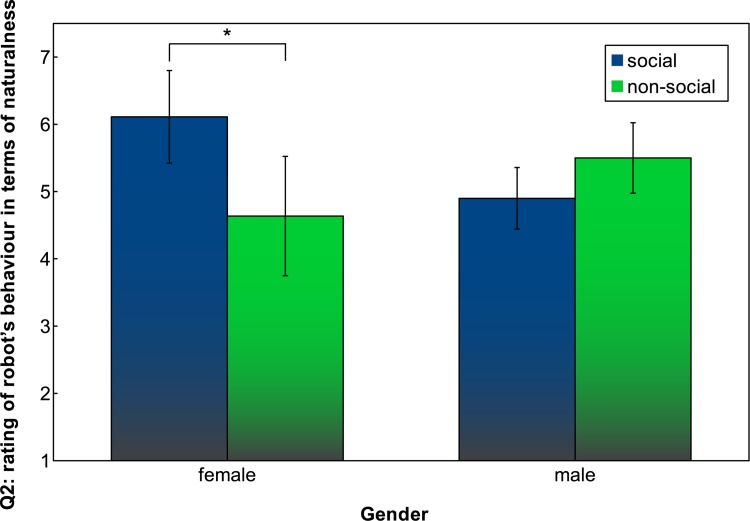
Q2: Participants’ rating of robot behaviour in terms of naturalness. Error bars indicate 95% confidence interval.

The questionnaire revealed some interactions between participants gender and the social-non social condition. For the question “How do you rate the robot’s behaviour?” (Q2), with a 7-point Likert scale ranging from ‘not natural at all’ to ‘very natural’, average ratings between the social and non-social condition hardly differ. However, when split into gender, a significant interaction between participants gender and the social condition can be observed (Fig 9). This is confirmed by an ANOVA with *F*(1, 34) = 8.4974, *p* = 0.006. The use of ANOVA to analyse Likert scale responses is appropriate here, as we use a 7-point Likert scale and sum at least 8 responses, thereby approaching normality and not requiring a non-parametric test [63]. No main effects were found, but an interaction exist: female participants find the robot’s behaviour in the social condition more natural than male participants, while it is the reverse in the non-social condition. A two sample t-test showed a significant difference in mean robot rating for female participants depending on the social condition, with *t*(17.664) = -2.5734, *p* = 0.0193, while this was not found for male participants (*t*(14.972) = 1.6912, *p* = 0.1115).

**Fig 10:**
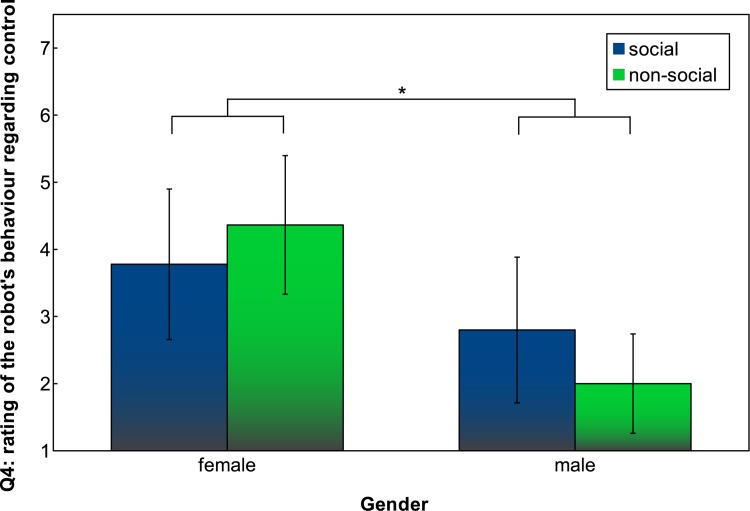
Q4: Participants’ rating of robot behaviour in terms of who was in control. Error bars indicate 95% confidence interval.

Answers to the question “Who was in control of the teaching sessions?” (Q4) [range: ‘I was in control’—‘the robot was in control’] indicated that female participants experienced the robot to be much more in control than male participants did. An ANOVA indicated a main effect on gender (*F*(1, 34) = 9.8195, *p* = 0.0035), but no interaction between gender and social/non-social condition was found (Fig 10).

**Fig 11:**
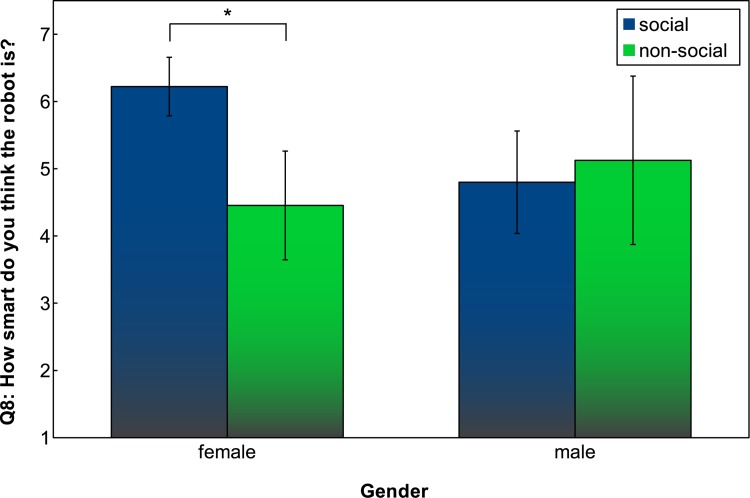
Q8: Participants’ rating of robot behaviour in terms of how smart the robot is. Error bars indicate 95% confidence interval.

For the question “How smart do you think the robot is?” (Q8) [range: ‘the robot is not smart at all’—‘the robot is very smart’], an ANOVA indicates a trend in the social/non-social condition (*F*(1, 34) = 3.2874, *p* = 0.0787) towards participants finding the robot smarter in the social condition. In addition, a clear interaction between gender and social/non-social can be observed (*F*(1, 34) = 5.9343, *p* = 0.0202); female participants judge the robot to be significantly smarter than male participants do in the social condition (Fig 11). A two sample t-test showed a significant difference in how smart the robot was found for female participants depending on the social condition, with *t*(15.059) = -3.7719, *p* = 0.0018, while this was not found for male participants (*t*(11.873) = 0.4345, *p* = 0.6717).

In summary, it appears that in the social condition female participants are more effective in teaching the robot, and in addition they rate the robot’s behaviour as more natural and the robot as smarter. These observed gender differences in perception and in the effectiveness of the robot’s social cues indicate that robots might have to adapt their social behaviour with respect to their interlocutor in order to maximise their performance.

## Discussion and Conclusion

We have presented an experiment in which a robot uses social cues during tutoring interactions with human teachers, which allowed the robot to modify and improve its learning input. In addition, in contrast with unstructured teaching, participants offer structured input which allow the robot to learn better, an effect that was more pronounced in the social condition. A similar effect is observed in child language acquisition: caregivers of preschool children seem to be aware of which conditions promote the successful acquisition of novel adjectives and will use linguistic utterances and referents that promote the speed and efficiency of lexical development [64].

This experiment serves as an illustration of how machine learning can be enhanced through embodiment in a social robot, resulting in more effective machine learning. Moreover, the findings illustrate how a robot might positively influence an interaction with a person through using social cues that are generally perceived as natural, as such sidestepping the need for specialised training on the user’s part.

While—in the social condition—the robot is quite explicit in expressing its preferences, this did not result in participants always adhering to this particular preference. What is clear, though, is that participants viewed the robot as an interaction partner for which they were motivated to tailor their teaching behaviour (as opposed to the unstructured offering of learning data). This emphasises the fact that, while people in general appear to be motivated to teach the robot, its behaviour needs to be carefully tuned to the situation, taking into account possible different objectives of the human interlocutor.

Clear gender differences were observed with respect to participants’ success in teaching the robot, and with respect to how participants experienced the robot subjectively. However, the reasons for why these findings are unknown. It may be that the robot’s (social) behaviour was interpreted differently by women and men. However, an analysis of participants’ personality tests did not reveal any significant interaction with other outcomes. As such, we conclude that participants’ personalities did not play a role in the findings reported here. One might speculate that the evolutionary history of the human species or the social role of gender might have an effect and how women and men tutor the robot, though our study did not aim to further elucidate this. Suffice to say that gender effects are often found in human-robot interaction: men and women frequently have different preferences or responses to robots [65–67] or respond differently when the robot is presented as having an overt gender [61].

The study provides a direction for developing social HRI and, more generally, machine learning, when artificial systems (such as robots) utilise social channels effectively to capitalise on human tutoring. The potential of this is substantial, as every person is naturally equipped with a sophisticated understanding of social cues when interacting with others. The main challenges in social machine learning are:

Social human learning is multi-modal in noisy environments Social human learning relies on the complex interplay of multi-modal exchanges—being both linguistic and paralinguistic in nature—in noisy environments, from the sensor level to the cognitive level. Creating open-ended artificial multi-modal communication is challenging; as such its application to machine learning has been limited.
Lack of understanding of social learning applied to machines While social learning in people is well studied [68–70], the transfer of social learning to artificial systems currently lacks a theoretical framework and, following from that, design guidelines on how social learning can be facilitated.
Learning input is alternatively structured Human social learning is different from machine learning, both in the nature of the learning experiences offered and accepted, and in the temporal structure of the learning [16, 71, 72]. As robots and computers benefit from structured and machine-readable input, dealing with experiences structured for human learning and the various social signals involved in tutoring is particularly challenging.

We believe these challenges set an agenda for the future of socially guided machine learning.

## Supporting Information

S1 File Spreadsheet containing all data in separate tabs. (ZIP)

S2 File Document containing the Questionnaire and BFI test. (PDF)
