# Supplementary material for: Why Robots Should Be Social: Enhancing Machine Learning through Social Human-Robot Interaction
Source: PLoS One. 2015 Sep 30;10(9):e0138061. doi: 10.1371/journal.pone.0138061 (PMC4589374; doi:10.1371/journal.pone.0138061)
Supplement: S2 File — (PDF) [file pone.0138061.s002.pdf]

# Social Robot Teaching Questionnaire

| | |
|---|---|
| **Participant number:** | **Age:** |
| **Gender: F / M** | **Native speaker: yes / no** |

**Please answer the following questions by placing an 'X' on the spot that best reflects your answer. Additionally, you can provide comments to elaborate your answers.**

**1. How do you rate your interaction with the robot?**

| | | | | | | |
|---|---|---|---|---|---|---|
| | | | | | | |

**not satisfactory at all** **very satisfactory**

**comments**

| |
|---|
| |

**2. How do you rate the robot's behaviour?**

| | | | | | | |
|---|---|---|---|---|---|---|
| | | | | | | |

**not natural at all** **very natural**

**comments**

| |
|---|
| |

**3. Do you have any experience with robots?**

| | | | | | | |
|---|---|---|---|---|---|---|
| | | | | | | |

**I have no experience with robots** **I have a lot of experience with robots**

**comments**

| |
|---|
| |

**4. Who was in control of the teaching sessions?**

| | | | | | | |
|---|---|---|---|---|---|---|
| | | | | | | |

**I was in control** **the robot was in control**

**comments**

| |
|---|
| |

**5. On what basis did you choose the animal examples as topic? Please explain.**

| |
|---|
| |

**6. Do you like science fiction (books, film, etc)?**

| | | | | | | |
|---|---|---|---|---|---|---|
| | | | | | | |

**I don't like science fiction at all** — **I very much like science fiction**

**comments**

| |
|---|
| |

**7. How many emotions do you think the robot has?**

| | | | | | | |
|---|---|---|---|---|---|---|
| | | | | | | |

**the robot has no emotions** — **the robot has a lot of emotions**

**comments**

| |
|---|
| |

**8. How smart do you think the robot is?**

| | | | | | | |
|---|---|---|---|---|---|---|
| | | | | | | |

**the robot is not smart at all** — **the robot is very smart**

**comments**

| |
|---|
| |

**9. How many hours per week do you spend using a computer?**

| |
|---|
| **hours computer use per week (estimate):** |

**comments**

| |
|---|
| |

**10. General comments**

| |
|---|
| |

**How I am in general**

Here are a number of characteristics that may or may not apply to you. For example, do you agree that you are someone who *likes to spend time with others*? Please write a number next to each statement to indicate the extent to which **you agree or disagree with that statement.**

| **1** Disagree Strongly | **2** Disagree a little | **3** Neither agree nor disagree | **4** Agree a little | **5** Agree strongly |
|---|---|---|---|---|

**I am someone who…**

1. _____ Is talkative
2. _____ Tends to find fault with others
3. _____ Does a thorough job
4. _____ Is depressed, blue
5. _____ Is original, comes up with new ideas
6. _____ Is reserved
7. _____ Is helpful and unselfish with others
8. _____ Can be somewhat careless
9. _____ Is relaxed, handles stress well
10. _____ Is curious about many different things
11. _____ Is full of energy
12. _____ Starts quarrels with others
13. _____ Is a reliable worker
14. _____ Can be tense
15. _____ Is ingenious, a deep thinker
16. _____ Generates a lot of enthusiasm
17. _____ Has a forgiving nature
18. _____ Tends to be disorganized
19. _____ Worries a lot
20. _____ Has an active imagination
21. _____ Tends to be quiet
22. _____ Is generally trusting
23. _____ Tends to be lazy
24. _____ Is emotionally stable, not easily upset
25. _____ Is inventive
26. _____ Has an assertive personality
27. _____ Can be cold and aloof
28. _____ Perseveres until the task is finished
29. _____ Can be moody
30. _____ Values artistic, aesthetic experiences
31. _____ Is sometimes shy, inhibited
32. _____ Is considerate and kind to almost everyone
33. _____ Does things efficiently
34. _____ Remains calm in tense situations
35. _____ Prefers work that is routine
36. _____ Is outgoing, sociable
37. _____ Is sometimes rude to others
38. _____ Makes plans and follows through with them
39. _____ Gets nervous easily
40. _____ Likes to reflect, play with ideas
41. _____ Has few artistic interests
42. _____ Likes to cooperate with others
43. _____ Is easily distracted
44. _____ Is sophisticated in art, music, or literature
